# Generation of two mode mechanical squeezing induced by nondegenerate parametric amplification

**DOI:** 10.1038/s41598-024-78168-x

**Published:** 2024-11-08

**Authors:** Muhdin Abdo Wodedo, Tesfay Gebremariam Tesfahannes, Tewodros Yirgashewa Darge, Mulgeta Taddese Bedore, Alemayehu Getahun Kumela, Gashaw Bekele Adera

**Affiliations:** 1 Department of Applied Physics, Adama Science and Technology University, 1888 Adama, Ethiopia; 2 Department of Physics, Arba Minch University, 21, Arba Minch, Ethiopia; 3 Department of Physics, Wachemo University, 667, Hossana, Ethiopia; 4 Department of Applied Physics, Mekdela Amba University, Tullu Awulia, Ethiopia; 5 Department of Physics, Haramaya University, 138 Dire Dawa, Ethiopia

**Keywords:** Physics, Quantum physics, Quantum information

## Abstract

Squeezing light in an optomechanical system involves reducing quantum noise in one of the light’s quadratures through the interaction between optical and mechanical modes. However, achieving successful implementation requires careful control of experimental parameters, which can be challenging. Here, we investigate a two-mode squeezed light transfer from optical to mechanical modes induced by a non-degenerate optical parametric amplifier (OPA). The optomechanical system is driven by frequencies nearly resonant with the anti-stokes fields that can realize cooling mechanical oscillators and quantum state transfer within a resolved sideband (good cavity) limit. Our results show that when a non-degenerate OPA is placed inside the optical cavity, the degree of squeezing in both optical and mechanical modes is significantly enhanced. This leads to the two-mode squeezed light being transferred into two-mode mechanical squeezing in the presence of the non-degenerate OPA under weak optomechanical coupling strength. Interestingly, we found that with negligible thermal bath noise, the two-mode squeezed light completely transferred to yield 50% mirror-mirror squeezing. In contrast, at higher thermal noise, the transfer of squeezed light is weak, causing the system to lose its quantum properties and behave more classically. Furthermore, we have shown that the degree of squeezing in the weak coupling regime drastically decreases with increasing mechanical dissipation rates. We believe that our scheme can achieve strong mechanical squeezing in hybrid optomechanical systems and facilitate homodyne detection to measure the quadratures of the squeezed light.

## Introduction

Cavity optomechanics is an exciting research field of study that explores the interaction between light (optical modes) and mechanical vibrations (mechanical modes) within a resonant cavity through radiation pressure^1–3^. In recent years, there has been a remarkable advancement in the discipline, with advancements in a variety of phenomena both fundamental research and practical applications^4^. Starting from cooling a nanomechanical resonator to multimode optomechanical systems^5–10^, several quantum features have been investigated, such as testing the quantum state of light^11–16^, transfer of entanglement from light-to-matter^17^, generation of bipartite and tripartite quantum correlations in mechanical oscillators^18^, generation of squeezing of the mechanical resonator^19–22^, optomechanically induced transparency^23^, quantum control of macroscopic mechanical systems^24^, exploiting profoundly various chiral topological effects^25^, experimental evidences in multimode optomechanical system^26,27^, and realizing quantum information processing^28–32^.

Recently, the properties of nonlinear mediums have become more essential to the quest to increase the accuracy and efficiency of sensitivity in measurements of hybrid systems. In this regard, squeezed light is a key resource for enhancing precision measurements and refers to quantum states with reduced noise along one of the quadrature components, and is the most valuable resource^33^. Moreover, it has recently been shown that the squeezing property of light is a physical resource used to reduce quantum noise^34^, sensing micromechanical displacement^35,36^, measurement-based quantum computation^37–39^, and detection of gravitational waves^40^. Furthermore, an interesting study^41–43^ experimentally realized that the reduction of noise below the shot-noise level is used to improve the performance of quantum key distribution systems, which effectively encode information and longer transmission distances improve^44^. This shows several intriguing uses due to their intrinsic non-linear optical characteristics, and the ability of light to squeeze itself has been extensively studied^45,46^. However, creating a portable and efficient squeezed light source has remained challenging. To overcome such a challenge, researchers introduce a non-linear medium element such as atomic gain medium^47^, Kerr material^48^, and optical parametric amplifier (OPA) inside a hybrid system^49^. Therefore, integrating nonlinear optics with OPA inside the cavity has paved an alternative path for improving quantum characteristics through their nonlinear interactions^50^. Considerable interest has recently been focused on optical nonlinearities as an excellent candidate for enhancing quantum effects in modern optical systems. Specifically, the degenerate optical parametric amplifier is among the simplest quantum optical systems and leads to several interesting applications. These applications include high-precision metrology^51^, measurement of weak classical forces^52^, quantum-enhanced measurements^53^, quantum information processing^54^, generation of degenerate squeezed light^55,56^, and experimentally realizing^57,58^.

Recently, squeezed light generation in a mechanical resonator garnered significant attention in quantum optomechanical systems. Accordingly, numerous schemes have been proposed to transfer the quantum features and archive strong squeezing^59–67^. Particularly, Agrawal et al.^68^ presented strong mechanical squeezing and detection in the presence of an OPA inside an optomechanical cavity. Moreover, Bai et al.^69^ report strong mechanical squeezing via the joint effect between Duffing non-linearity and parametric pumping. In a study by Huang et al.^70^, a controllable squeezed state of each of the two mechanical modes in an optomechanical system has been realized by breaking the dark-mode effect with synthetic magnetism and parametric pumping. Those findings suggested that it is possible to obtain noise-resistant quantum resources. In another study, Bekele et al.^71^ describe a robust two-mode mechanical squeezing utilizing three-level atoms in a doubly resonant optomechanical cavity. Moreover, Ahmed et al.^72^ have suggested an enhancement of cavity-mirror entanglement via non-degenerate OPA in a bimodal optomechanical cavity with a single movable mirror. In addition, Luo et al.^73^ design a scheme of quantum feedback mechanism to a doubly resonant optomechanical cavity with non-degenerate parametric down-conversion (NPD). They reported theoretically an optimal two-mode mechanical squeezing of 3 dB when the feedback mechanism is turned off near the threshold pumping strength of the laser field to NPD. Furthermore, they found that the feedback mechanism aids in beating the mechanical squeezing of the 3 dB limit. Motivated by recent interest in squeezed light generation, an interesting question is whether a scheme of doubly resonant cavities with a non-degenerate OPA inside can transfer the quantum feature of squeezed cavity fields to its coupled two movable mirrors through radiation pressure. This question is motivated by the recent interest in squeezed state generation and multi-mode investigations of light squeezing enhancement by coupling nonlinear optical cavities.

This paper proposes a two-mode squeezed light transfer scheme to nano-mechanical oscillators induced by a non-degenerate OPA. Specifically, we have considered a hybrid optomechanical system that consists of non-degenerate OPA inside a doubly resonant cavity coupled with two mechanical oscillators through radiation pressure. Particularly, we focus on the impact of non-degenerate OPA that governs the degree of squeezing in mechanical oscillators. In addition, the system under our investigation performs in red-detuning of laser drives that enhances the cooling efficiency of mechanical oscillators and facilitates quantum state transfer in the resolved sideband regime. Furthermore, the optical squeezing is transferred to the mechanical modes utilizing non-degenerate OPA under the hybrid optomechanical system. Accordingly, we show that the squeezing is phase-sensitive due to the involvement of non-degenerate OPA. Furthermore, the degree of squeezing in cavity fields and mechanical modes exhibits noise suppression at a particular parametric gain corresponding to each selected set of parametric phases. Moreover, with a choice of parameters related to non-degenerate OPA and weak coupling, the squeezing between the two-mode cavity fields is more robust. However, the robustness of the squeezing between two movable mirrors is obtained when the coupling becomes stronger. The squeezing in nano-mechanical oscillators is negatively affected by thermal noise. Therefore, the present result of squeezing in mechanical oscillators can be easily detected when quantum state transfer is induced. Furthermore, we anticipate that this approach holds promise for applications in quantum sensing and quantum information processing.

The other parts of this paper are presented in the following manner. In Section “Model and dynamics of motion”, we introduce the model and the Hamiltonian of the hybrid optomechanical system, which consists of non-degenerate OPA. We also obtain the quantum Langevin equations for the Hamiltonian of the system, including steady-state solutions and the linearization of quantum fluctuation equations under rotating wave approximation. In Section “Optomechanical two-mode quadrature squeezing light”, we provide analytical expressions to quantify two-mode squeezing in cavity modes and mechanical modes via utilizing the numerical solutions of the covariance matrix elements in the Lyapunov equation. In this section, we also discuss the numerical results under the effects of various parameters on two-mode squeezing. In Section “Detection of two-mode mechanical squeezing”, the experimental realization of the detection of two-mode mechanical squeezing is briefly discussed. The conclusions of our findings are summarized in Section “Conclusions”.

## Model and dynamics of motion

**Fig. 1:**
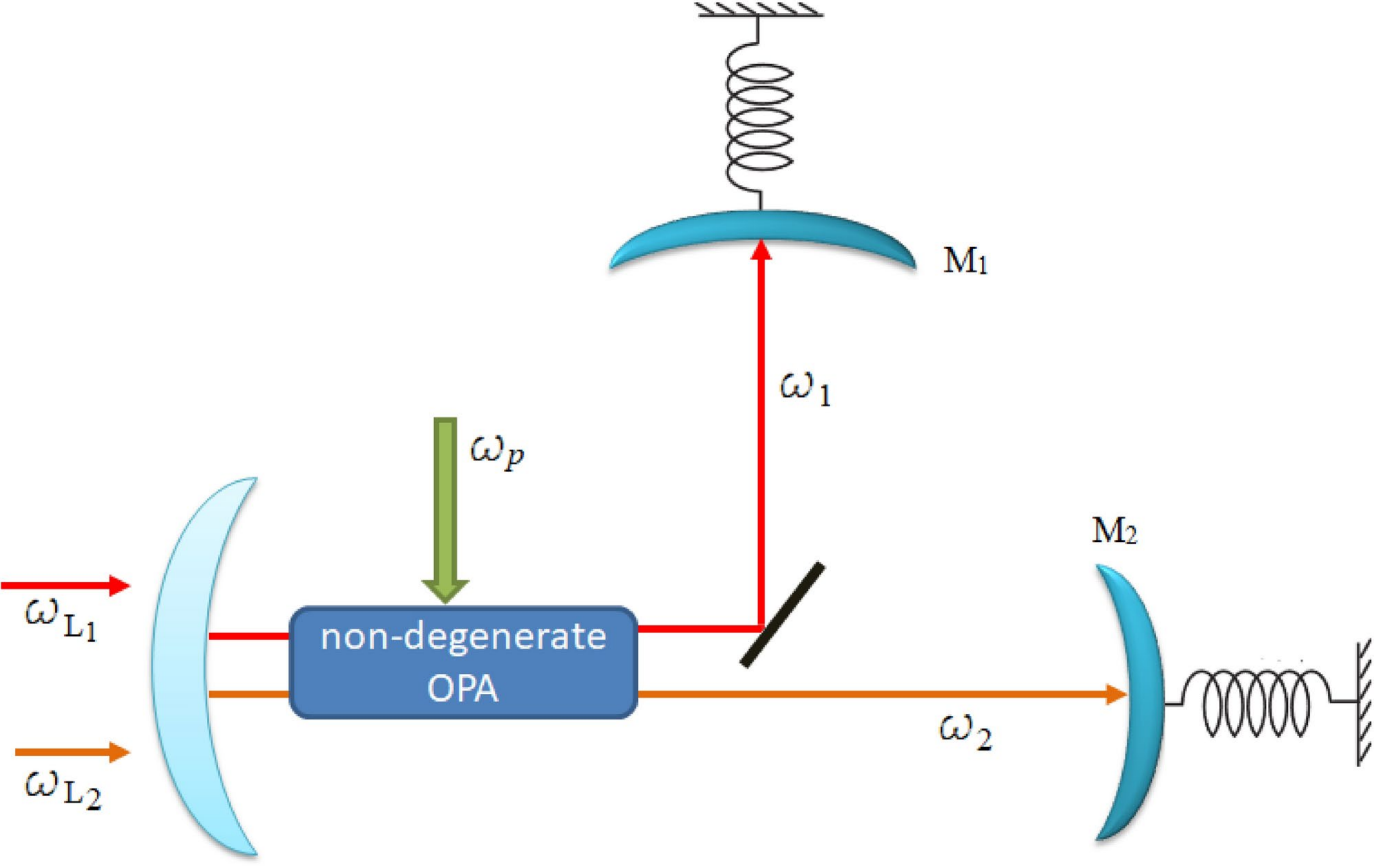
Schematic diagram of a doubly resonant optomechanical cavity having one fixed mirror and two movable mirrors, $M_1$ and $M_2$. The cavity contains a non-degenerate optical parametric amplifier pumped by $\omega _{p}$. The cavity is driven by input coherent laser fields with frequencies $\omega _{L_1}$ and $\omega _{L_2}$. The other parameters are defined in the main text.

We consider a hybrid optomechanical system consisting of two optical modes and two mechanical modes, as shown in Fig. 1. The schematic model consists of a pumped non-degenerate OPA within a doubly resonant Fabry-Pérot-type cavity with a fixed mirror and two completely reflecting mechanical mirrors ($M_1$ and $M_2$). Here, the non-degenerate OPA is usually used to generate two-mode squeezed states. Thus, the movable mirror experiences radiation pressure from the intracavity photons, which results in an optomechanical interaction between the movable mirror and every cavity field. Each cavity field with resonance frequency $\omega _{j}$ is driven by an external laser with frequency $\omega _{L_j}$ and amplitude $\varepsilon _{j}$. Thus, the movable mirror experiences radiation pressure from the intracavity photons, which results in an optomechanical interaction between the movable mirror and every cavity field. The movable mirrors are in equilibrium with their respective thermal baths, causing a thermal Langevin force to act on each one of the mirrors. Furthermore, each mirror experiences small oscillations around its equilibrium position due to the combined effect of these two forces. The movable mirrors are regarded as quantum-mechanical harmonic oscillators, with an effective mass of $m_j$, a frequency of $\omega _{m_j}$, and an energy decay rate of $\gamma _{m_j}$. To this aim, we consider that the non-linear parametric gain ($G_a$) of the non-degenerate OPA is directly proportional to the power of the pump driving the non-degenerate OPA, and the parametric phase of the pump field for the non-degenerate OPA is $\phi$. We assume the non-degenerate OPA is pumped at frequency $\omega _{p}= \sum _{j=1}^{2}(\omega _{L_j}+\omega _{m_j})$ and interacts with a second-order nonlinear optical crystal, thus it is down-converted to signal and idler photons with frequency $\omega _{j}=\omega _{L_j}+\omega _{m_j}$.

The total Hamiltonian of the system can be written as ($\hbar =1$): ${\hat{H}} = {\hat{H}}_0+{\hat{H}}_1$ with

1 $$\begin{aligned} \begin{aligned} {\hat{H}}_0&=\sum ^{2}_{j=1}\big [\omega _{j}{\hat{a}}^\dagger _{j} {\hat{a}}_{j} + \omega _{m_j}{\hat{b}}^\dagger _{j}{\hat{b}}_{j} - g_{j} {\hat{a}}^\dagger _{j}{\hat{a}}_{j}({\hat{b}}^\dagger _{j}+{\hat{b}}_{j}) +i\big (\varepsilon _{j}{\hat{a}}^\dagger _{j}e^{-i\omega _{L_j}t}-\varepsilon ^*_{j}{\hat{a}}_{j}e^{i\omega _{L_j}t}\big )\big ], \\ {\hat{H}}_1&=i G_a \big (e^{i\phi } {\hat{a}}^\dagger _{1}{\hat{a}}^\dagger _{2}e^{-i\omega _{p}t}-e^{-i\phi }{\hat{a}}_{1}{\hat{a}}_{2}e^{i\omega _{p}t}\big ), \end{aligned} \end{aligned}$$

where the first and second terms in the first line indicate the Hamiltonian of the two cavity modes and the mechanical modes, respectively, with ${\hat{a}}_{j}({\hat{b}}_{j})$ and ${\hat{a}}^\dagger _{j}({\hat{b}}^\dagger _{j})$ are annihilation and creation operators of the cavity (mechanical) that satisfy the commutation relation, $[{\hat{a}}_{j},{\hat{a}}_{j'}^{\dagger }] =[{\hat{b}}_{j},{\hat{b}}_{j'}^{\dagger }]=\delta _{jj'}$. The third term in the first line describes the interaction Hamiltonian of the cavity modes and mechanical modes with coupling $g_{j}=\frac{\omega _{j}}{L_{j}}\sqrt{\frac{\hbar }{2m_j\omega _{m_j}}}$ is a single-photon-level optomechanical strength with $L_{j}$ as the length of each cavity. The fourth term in the first line represents the interaction Hamiltonian of the coherent laser fields with the cavity modes, with $|\varepsilon _{j}|=\sqrt{\frac{\kappa _{j}P_{j}}{\hbar \omega _{L_j}}}$ where $P_{j}$ is the power of the laser drive and rate $\kappa _{j}$ is the cavity decay due to the dissipation of photons through the partially transmitting fixed mirror. The first term in the second line is a prominent Hamiltonian due to the coupling (interaction) of cavity modes with non-degenerate OPA. Accordingly, the Hamiltonian of the system in a rotating frame is defined by the unitary transformation operator, $e^{-it(\omega _{L_1}\hat{a}^\dagger _{1}{\hat{a}}_{1}+\omega _{L_2}\hat{a}^\dagger _{2}{\hat{a}}_{2})}$, becomes

2 $$\begin{aligned} {\hat{H}}= & \sum ^{2}_{j=1} \big [\Delta '_{j} \hat{a}^\dagger _{j}{\hat{a}}_{j} + \omega _{m_j}{\hat{b}}^\dagger _{j}{\hat{b}}_{j} - g_{j} {\hat{a}}^\dagger _{j}{\hat{a}}_{j}({\hat{b}}^\dagger _{j}+{\hat{b}}_{j}) +i\big (\varepsilon _{j}{\hat{a}}^\dagger _{j}-\varepsilon ^*_{j}{\hat{a}}_{j}\big )\big ] +i G_a \big (e^{i\phi } {\hat{a}}^\dagger _{1}{\hat{a}}^\dagger _{2}e^{-i(\omega _{m_1}+\omega _{m_2})t}\nonumber \\ & -e^{-i\phi } {\hat{a}}_{1}{\hat{a}}_{2}e^{i(\omega _{m_1}+\omega _{m_2})t}\big ), \end{aligned}$$

in which $\Delta '_{j}=\omega _{j}-\omega _{L_j}$ is cavity detuning.

We can derive the dynamical equations that govern the system by applying the Heisenberg-Langevin equation that considers quantum and thermal noises. Therefore, based on the Hamiltonian in Eq. (2), we can obtain the following equations of motion for the two mechanical modes and two optical modes as

3 $$\begin{aligned} \begin{aligned} \dot{{\hat{b}}}_{1}&=-i\omega _{m}{\hat{b}}_{1}+ ig_{1}{\hat{a}}^\dagger _{1}{\hat{a}}_{1} - \frac{\gamma _{m}}{2}{\hat{b}}_{1}+ \sqrt{\gamma _{m}}\ {\hat{b}}^{in}_{1}, \\ \dot{{\hat{b}}}_{2}&=-i\omega _{m}{\hat{b}}_{2}+ ig_{2}{\hat{a}}^\dagger _{2}{\hat{a}}_{2} - \frac{\gamma _{m}}{2}{\hat{b}}_{2}+ \sqrt{\gamma _{m}}\ {\hat{b}}^{in}_{2}, \\ \dot{{\hat{a}}}_{1}&=-i\Delta '_{1}{\hat{a}}_{1}+ig_{1}{\hat{a}}_{1}({\hat{b}}^\dagger _{1}+{\hat{b}}_{1})+\varepsilon _{1} + G_ae^{i\phi }e^{-2i\omega _{m}t}{\hat{a}}^\dagger _{2}-\frac{\kappa }{2}{\hat{a}}_{1}+\sqrt{\kappa }{\hat{a}}^{in}_{1}, \\ \dot{{\hat{a}}}_{2}&=-i\Delta '_{2}{\hat{a}}_{2}+ig_{2}{\hat{a}}_{2}({\hat{b}}^\dagger _{2}+{\hat{b}}_{2})+\varepsilon _{2} + G_ae^{i\phi }e^{-2i\omega _{m}t}{\hat{a}}^\dagger _{1}-\frac{\kappa }{2}{\hat{a}}_{2}+\sqrt{\kappa }{\hat{a}}^{in}_{2}, \end{aligned} \end{aligned}$$

where it is assumed that the two mirrors have the same mechanical frequency $\omega _{m}$ and dissipation rate $\gamma _{m}$, and the optical mode of each cavity decay rate is $\kappa$. Furthermore, the ${\hat{a}}^{in}_{j}$ and ${\hat{b}}^{in}_{j}$ are the input noise operators associated with the $j^{th}$ optical mode and the $j^{th}$ mechanical mode, respectively. Assuming the noise operator of the mirrors operating at ultra-low temperatures as a Gaussian white-noise source and considering high-frequency mechanical oscillators $\omega _{m}$ leads to a high mechanical quality factor $Q_{m}=\omega _{m}/\gamma _{m}\gg 1$. This shows that the evolution of mechanical resonators undergoes the Markovian process^74^. Thus, for the thermal noise operator ${\hat{b}}^{in}_{j}$ with zero mean value and its nonzero delta correlation functions^75^ and given by $\big <{\hat{b}}^{in,\dagger }_{j}(t){\hat{b}}^{in}_{j}(t')\big >=n_{j}\delta (t-t')$ and $\big <{\hat{b}}^{in}_{j}(t){\hat{b}}^{in,\dagger }_{j}(t')\big > =(n_{j}+1)\delta (t-t')$ with $n_{j}=\big [e^{\hbar \omega _{m} /(k_B T_{j})}-1\big ] ^{-1}$ is the initial mean thermal excitation number in the $j^{th}$ movable mirror at temperature $T_{j}$, and $k_{B}$ denotes the standard Boltzmann’s constant. In addition, the quantum (vacuum) noise operator ${\hat{a}}^{in}_{j}$ also has a zero mean value, and its nonzero correlation function^76^ takes the form of $\big <{\hat{a}}^{in}_{j}(t){\hat{a}}^{in,\dagger }_{j}(t')\big >=\delta (t-t')$.

Quantifying steady-state squeezing is particularly relevant, which occurs when the cavity is strongly driven, resulting in a highly significant intracavity field^77^. Under this condition, the dynamics of the system can be expanded as a form of c-number of a steady-state value of the operator plus a quantum fluctuation operator having zero average, written as ${\hat{a}}_{j} = \alpha _{j}+\delta {\hat{a}}_{j}$ and ${\hat{b}}_{j} = \beta _{j}+\delta {\hat{b}}_{j}$. Accordingly, the steady-state solutions are simplified as

4 $$\begin{aligned} \alpha _{j}&=\frac{\varepsilon _{j}}{\frac{\kappa }{2}+i\Delta _{j}},&\beta _{j}&=\frac{i g_{j}|\alpha _{j}|^2 }{\frac{\gamma _{m}}{2}+i\omega _{m}}, \end{aligned}$$

for $\Delta _{j}=\Delta '_{j}-g_{j}(\beta _{j}+\beta _{j}^{*})$ is the effective cavity-laser field detuning of the cavity. Here $\alpha _{j}$ is the $j^{th}$ cavity mode steady-state amplitude of the cavity field, and the $j^{th}$ mechanical mode $\beta _{j}$ determines the steady-state displacement of the movable mirror. The average numbers of photons and phonons at steady state corresponding to each cavity and mechanical modes are given by $|\alpha _{j}|^2$ and $|\beta _{j}|^2$, respectively. The parameter regime that matters for generating significant quantum correlations is when $|\alpha _{j}|\gg 1$, or when the input power $P_j$ is high. In this instance, the linearized Langevin equations can be obtained by safely neglecting the higher-order terms: $\delta {\hat{a}}^\dagger _{1}\delta {\hat{a}}_{1}$, $\delta {\hat{a}}^\dagger _{2}\delta {\hat{a}}_{2}$, $\delta {\hat{a}}_{1}(\delta {\hat{b}}^\dagger _{1}+\delta {\hat{b}}_{1})$, and $\delta {\hat{a}}_{2}(\delta {\hat{b}}^\dagger _{2}+\delta {\hat{b}}_{2})$ in the quantum fluctuation dynamics equations and Eq.(3) can be linearized as

5 $$\begin{aligned} \begin{aligned} \delta \dot{{\hat{b}}}_{1}&=-\big (\frac{\gamma _{m}}{2}+i\omega _{m}\big )\delta {\hat{b}}_1 +ig_{1}(\alpha _{1}\delta {\hat{a}}^\dagger _{1}+\alpha ^*_{1}\delta {\hat{a}}_{1})+\sqrt{\gamma _{m}}{\hat{b}}^{in}_{1},\\ \delta \dot{{\hat{b}}}_{2}&=-\big (\frac{\gamma _{m}}{2}+i\omega _{m}\big )\delta {\hat{b}}_2 +ig_{2}(\alpha _{2}\delta {\hat{a}}^\dagger _{2}+\alpha ^*_{2}\delta {\hat{a}}_{2})+\sqrt{\gamma _{m}}{\hat{b}}^{in}_{2},\\ \delta \dot{{\hat{a}}}_{1}&= -\big (\frac{\kappa }{2}+i\Delta _{1}\big )\delta {\hat{a}}_{1}+ig_{1}\alpha _1(\delta {\hat{b}}^\dagger _{1} +\delta {\hat{b}}_{1})+ G_ae^{i\phi }e^{-2i\omega _{m}t}\delta {\hat{a}}^\dagger _{2} +\sqrt{\kappa }{\hat{a}}^{in}_{1}, \\ \delta \dot{{\hat{a}}}_{2}&= -\big (\frac{\kappa }{2}+i\Delta _{2}\big )\delta {\hat{a}}_{2}+ig_{2}\alpha _2(\delta {\hat{b}}^\dagger _{2} +\delta {\hat{b}}_{2})+ G_ae^{i\phi }e^{-2i\omega _{m}t}\delta {\hat{a}}^\dagger _{1} +\sqrt{\kappa }{\hat{a}}^{in}_{2}. \end{aligned} \end{aligned}$$

Furthermore, we introduce slowly varying operators $\delta {\tilde{a}}_{j}=\delta {\hat{a}}_{j}e^{i\Delta _{j} t}, {\tilde{a}}^{in}_{j}={\hat{a}}^{in}_{j}e^{i\Delta _{j} t}$, $\delta {\tilde{b}}_{j}=\delta {\hat{b}}_{j}e^{i\omega _{m} t}$ and ${\tilde{b}}^{in}_{j}={\hat{b}}^{in}_{j}e^{i\omega _{m} t}$ into Eq.(5) and the dynamics of quantum fluctuations becomes

6 $$\begin{aligned} \begin{aligned} \delta \dot{{\tilde{b}}}_{1}&=-\frac{\gamma _{m}}{2}\delta {\tilde{b}}_1 +iG_{1}\big (\delta {\tilde{a}}^\dagger _{1}e^{i(\Delta _{1}+\omega _{m})t}+\delta {\tilde{a}}_{1}e^{-i(\Delta _{1}-\omega _{m})t}\big )+\sqrt{\gamma _{m}}{\tilde{b}}^{in}_{1},\\ \delta \dot{{\tilde{b}}}_{2}&=-\frac{\gamma _{m}}{2}\delta {\tilde{b}}_2 +iG_{2}\big (\delta {\tilde{a}}^\dagger _{2}e^{i(\Delta _{2}+\omega _{m})t}+\delta {\tilde{a}}_{2}e^{-i(\Delta _{2}-\omega _{m})t}\big )+\sqrt{\gamma _{m}}{\tilde{b}}^{in}_{2},\\ \delta \dot{{\tilde{a}}}_{1}&= -\frac{\kappa }{2}\delta {\tilde{a}}_{j}+iG_{1}\big (\delta {\tilde{b}}^\dagger _{1} e^{i(\Delta _{1}+\omega _{m}) t}+\delta {\tilde{b}}_{1} e^{i(\Delta _{1}-\omega _{m}) t}\big )+ G_ae^{i\phi }e^{i(\Delta _{1}-\omega _{m})t}e^{i(\Delta _{2}-\omega _{m})t}\delta {\tilde{a}}^\dagger _{2} +\sqrt{\kappa }{\tilde{a}}^{in}_{1}, \\ \delta \dot{{\tilde{a}}}_{2}&= -\frac{\kappa }{2}\delta {\tilde{a}}_{2}+iG_{2}\big (\delta {\tilde{b}}^\dagger _{2} e^{i(\Delta _{2}+\omega _{m}) t}+\delta {\tilde{b}}_{2} e^{i(\Delta _{2}-\omega _{m}) t}\big )+ G_ae^{i\phi }e^{i(\Delta _{2}-\omega _{m})t}e^{i(\Delta _{1}-\omega _{m})t}\delta {\tilde{a}}^\dagger _{1} +\sqrt{\kappa }{\tilde{a}}^{in}_{2}, \end{aligned} \end{aligned}$$

where $G_{j}=g_{j} |\alpha _{j}|$ is the effective optomechanical coupling rate that depends on the power $P_{j}$ of the input laser. By assuming the laser field amplitude $\varepsilon _{j}= |\varepsilon _{j}|e^{ -i\varphi _{j}}$, and $\varphi _{j}$ represents the phase of the laser input field coupling to the optical field, we choose $\varphi _{j}$ to satisfy $e^{-i\varphi _{j}} =(\kappa /2+i\Delta _{j}) \big / \sqrt{(\kappa /2)^2+\Delta ^2_{j}}$ such that $\alpha _{j}$ can be real^78^. Since the driving field is thought to be red-detuned from the cavity resonance ($\Delta _{1}=\Delta _{2}=\omega _{m}$), the anti-Stokes scattered light is almost resonant with the cavity field. Furthermore, we assume that the mechanical quality factor is high ($\omega _{m} \gg \gamma _{m}$), the mechanical frequency $\omega _{m}$ is significantly bigger than $G_j$ and $G_a$, while the system is operating in the resolved sideband (good cavity limit) regime, $\omega _{m}\gg \kappa$. Under these scenarios, it is feasible to apply the rotating wave approximation to omit the fast oscillating terms $e^{2i\omega _mt}$ in Eq. (6), resulting in

7 $$\begin{aligned} \begin{aligned} \delta \dot{{\tilde{b}}}_{1}&= -\frac{\gamma _{m}}{2}\delta {\tilde{b}}_1 +iG_{1}\delta {\tilde{a}}_{1} +\sqrt{\gamma _{m}} {\tilde{b}}^{in}_{1}, \\ \delta \dot{{\tilde{b}}}_{2}&= -\frac{\gamma _{m}}{2}\delta {\tilde{b}}_2 +iG_{2}\delta {\tilde{a}}_{2} +\sqrt{\gamma _{m}} {\tilde{b}}^{in}_{2}, \\ \delta \dot{{\tilde{a}}}_{1}&= -\frac{\kappa }{2}\delta {\tilde{a}}_{1}+iG_{1}\delta {\tilde{b}}_{1} + G_ae^{i\phi }\delta {\tilde{a}}^\dagger _{2} +\sqrt{\kappa }{\tilde{a}}^{in}_{1} \\ \delta \dot{{\tilde{a}}}_{2}&= -\frac{\kappa }{2}\delta {\tilde{a}}_{2}+iG_{2}\delta {\tilde{b}}_{2} + G_ae^{i\phi }\delta {\tilde{a}}^\dagger _{1} +\sqrt{\kappa }{\tilde{a}}^{in}_{1}. \end{aligned} \end{aligned}$$

We can see in Eq. (7), the mechanical mode and cavity mode undergo beam-splitter-like interaction, whereas the cavity modes have parametric down conversion type interaction. Thus, the squeezing of the two-mode fields can be transferred to the squeezing of movable mirrors.

We introduce dimensionless Hermitian quadrature fluctuating operators with the corresponding noise operators to quantify two-mode squeezing in optical and mechanical modes. Thus, the cavity mode amplitude and phase are defined as $\delta \tilde{x}_{j}=\frac{1}{\sqrt{2}}(\delta {\tilde{a}}^\dagger _{j}+\delta {\tilde{a}}_{j})$ and $\delta \tilde{y}_{j}=\frac{i}{\sqrt{2}}(\delta {\tilde{a}}^\dagger _{j}-\delta {\tilde{a}}_{j})$ with their corresponding input quantum noise quadrature operators as ${\tilde{x}}^{in}_{j}=\frac{1}{\sqrt{2}}({\tilde{a}}^{in,\dagger }_{j}+{\tilde{a}}^{in}_{j})$ and ${\tilde{y}}^{in}_{j}=\frac{i}{\sqrt{2}}({\tilde{a}}^{in,\dagger }_{j}-{\tilde{a}}^{in}_{j})$. Similarly, we define the mechanical mode position and momentum quadrature operators as $\delta \tilde{q}_{j}=\frac{1}{\sqrt{2}}(\delta {\tilde{b}}^\dagger _{j}+\delta {\tilde{b}}_{j})$ and $\delta \tilde{p}_{j}=\frac{i}{\sqrt{2}}(\delta {\tilde{b}}^\dagger _{j} -\delta {\tilde{b}}_{j})$, with their corresponding input thermal noise quadrature operators introduced as ${\tilde{q}}^{in}_{j}=\frac{1}{\sqrt{2}}({\tilde{b}}^{in,\dagger }_{j}+{\tilde{b}}^{in}_{j})$ and ${\tilde{p}}^{in}_{j}=\frac{i}{\sqrt{2}}({\tilde{b}}^{in,\dagger }_{j}-{\tilde{b}}^{in}_{j})$. Using those quadrature fluctuation operators, Eq. (7) can be written as a first-order differential equation in compact matrix form as

8 $$\begin{aligned} \frac{d}{dt}{{\textbf {u}}(t)}= {\textbf {J}} {\textbf {u}}(t)+{\textbf {v}}(t), \end{aligned}$$

where ${\textbf {u}}(t)=(\delta {{\tilde{q}}}_{1},\delta {\tilde{p}}_{1}, \delta {{\tilde{q}}}_{2},\delta {{\tilde{p}}}_{2}, \delta {{\tilde{x}}}_{1},\delta {{\tilde{y}}}_{1},\delta {\tilde{x}}_{2},\delta {{\tilde{y}}}_{2}) ^T$ is the vector of quadrature fluctuation operators, ${\textbf {J}}$ is the drift matrix which contains information about the system given by

9 $$\begin{aligned} {\textbf {J}}=\begin{bmatrix} -\frac{\gamma _{m}}{2} & 0 & 0 & 0& 0 & -G& 0 & 0\\ 0 & -\frac{\gamma _{m}}{2}& 0 & 0 & G & 0 & 0 & 0\\ 0 & 0 & -\frac{\gamma _{m}}{2} & 0 & 0 & 0& 0 & -G\\ 0& 0 & 0 & -\frac{\gamma _{m}}{2}& 0& 0& G & 0\\ 0 & -G & 0 & 0& -\frac{\kappa }{2} & 0& G_a\cos {\phi } & G_a\sin {\phi }\\ G & 0& 0 & 0& 0& -\frac{\kappa }{2}& G_a\sin {\phi } & -G_a\cos {\phi }\\ 0& 0 & 0 & -G& G_a \cos {\phi } & G_a \sin {\phi }& -\frac{\kappa }{2}& 0 \\ 0 & 0 & G & 0& G_a\sin {\phi }& -G_a\cos {\phi }& 0 & -\frac{\kappa }{2}\\ \end{bmatrix}, \end{aligned}$$

with $G=G_1= G_2$, and ${\textbf {v}}(t) = (\sqrt{\gamma _{m}}{\tilde{q}}^{in}_{1}, \sqrt{\gamma _{m}}{\tilde{p}}^{in}_{1}, \sqrt{\gamma _{m}}{\tilde{q}}^{in}_{2} \sqrt{\gamma _{m}}{\tilde{p}}^{in}_{2}, \sqrt{\kappa }{\tilde{x}}^{in}_{1}, \sqrt{\kappa }{\tilde{y}}^{in}_{1}, \sqrt{\kappa }{\tilde{x}}^{in}_{2}, \sqrt{\kappa }{\tilde{y}}^{in}_{2}) ^T$ is the vector of the noise sources.

To study the stability conditions of the system, we have to consider the steady-state scenario defined by Eq. (8). Particularly, the stability condition of the system can be achieved by applying the Routh-Hurwitz stability criterion^79^ that confirms all the eigenvalues of the drift matrix ${\textbf {J}}$ have negative real parts. Accordingly, we obtain three independent stability conditions for the system, given as follows:

10 $$\begin{aligned} c_1= &\,(\kappa +\gamma _m)\big [\kappa \gamma _m+\frac{1}{4}(\kappa ^2+\gamma ^2_m)+2{G}^2-G^2_a\big ]-\big [(\kappa +\gamma _m){G}^2+\frac{\gamma _m}{4}(\gamma _m\kappa +\kappa ^2-4G^2_a)\big ]> 0,\nonumber \\ c_2= &\,(\kappa +\gamma _m)\big [\kappa \gamma _m+\frac{1}{4}(\kappa ^2+\gamma ^2_m)+2{G}^2-G^2_a\big ]\big [(\kappa +\gamma _m){G}^2+\frac{\gamma _m}{4}(\gamma _m\kappa +\kappa ^2-4G^2_a)\big ]\nonumber \\ & -\frac{\gamma _m}{2}\bigg [\frac{1}{8}\gamma _m(\kappa ^2-4G^2_a)+\kappa {G}^2\bigg ](\kappa +\gamma _m)^2 -\big [(\kappa +\gamma _m){G}^2+\frac{\gamma _m}{4}(\gamma _m\kappa +\kappa ^2-4G^2_a)\big ]^2> 0,\nonumber \\ c_3= &\,\frac{1}{16}\gamma ^2_m(\kappa ^2-4G^2_a)+\frac{1}{2}\gamma _m\kappa {G}^2 > 0. \end{aligned}$$

When we set $G=0$ in the last condition $c_3>0$, the system shows stationary features in a regime only if $G_a < 0.5\kappa$, while the stability conditions are independent of the parametric phase $\phi$. In order to study the generation of two-mode squeezing in the system, we calculate the steady-state value of the covariance matrix, which is fully characterized by an $8\times 8$ covariance matrix with corresponding matrix elements as

11 $$\begin{aligned} V_{ll'} =\frac{1}{2} \big [\big<u_l(\infty ) u_{l'}(\infty )\big>+\big <u_{l'}(\infty ) u_l(\infty )\big >\big ], \ \ (l,l' = 1, 2, 3,..., 8) \end{aligned}$$

with ${\textbf {u}}(\infty )=\big (\delta {\tilde{q}}_{1}(\infty ),\delta {{\tilde{p}}}_{1}(\infty ),\delta {\tilde{q}}_{2}(\infty ),\delta {{\tilde{p}}}_{2}(\infty ), \delta {\tilde{x}}_{1}(\infty ),\delta {{\tilde{y}}}_{1}(\infty ),\delta {\tilde{x}}_{2}(\infty ),\delta {{\tilde{y}}}_{2}(\infty )\big ) ^T$. For a stable system in steady state, we can derive the covariance matrix ${\textbf {V}}$ using the Lyapunov equation^80^,

12 $$\begin{aligned} {\textbf {JV}}+ {\textbf {VJ}}^T=-{\textbf {D}}, \end{aligned}$$

where ${\textbf {D}}$ is the diffusion matrix whose elements can be obtained from the elements of the column vector of the noise sources and making use of the input noise correlations of the cavity and mechanical modes in $D_{ll'} \delta (t-t')=\frac{1}{2}\big [\big<v_l(t) v_{l'}(t')\big>+\big <v_{l'}(t') v_l(t)\big >\big ]$. Consequently, we obtain a diagonal diffusion matrix,

13 $$\begin{aligned} {\textbf {D}}=Diag \left[ \frac{\gamma _{m}}{2}(2n+1),\frac{\gamma _{m}}{2}(2n+1),\frac{\gamma _{m}}{2}(2n+1),\frac{\gamma _{m}}{2}(2n+1),\frac{\kappa }{2},\frac{\kappa }{2},\frac{\kappa }{2},\frac{\kappa }{2}\right] . \end{aligned}$$

Since the quantum noises (${\tilde{x}}^{in}_{j}, {\tilde{y}}^{in}_{j}$) as well as the thermal noises (${\tilde{q}}^{in}_{j}, {\tilde{p}}^{in}_{j}$) are all zero-mean Gaussian and the dynamics of the system have been linearized, the steady state of the system goes to a zero-mean multipartite Gaussian state.

## Optomechanical two-mode quadrature squeezing light

In this section, we measure the two-mode squeezed state optical fields and the transfer of optical fields to nano-mechanical oscillators induced by a non-degenerate OPA. Thus, we quantify the two-mode quadrature squeezing of optical and mechanical modes under various parameters. To analyze the two-mode optomechanical squeezing, we utilize the collective quadrature pairs of position and momentum operators with a zero mean defined by $Q_{\pm }=\delta {\tilde{q}}_{1}\pm \delta {\tilde{q}}_{2}$ and $P_{\pm }=\delta {\tilde{p}}_{1}{\pm }\delta {\tilde{p}}_{2}$ for mechanical modes. Similarly, for cavity modes, the collective quadrature pairs of amplitude and phase operators are defined as $X_{\pm }=\delta {\tilde{x}}_{1}{\pm }\delta {\tilde{x}}_{2}$ and $Y_{\pm }=\delta {\tilde{y}}_{1}{\pm }\delta {\tilde{y}}_{2}$. Thus, the quadrature variances can be expressed simply as

14 $$\begin{aligned} \begin{aligned} \big<Q_{\pm }^2\big>&=\big<\delta {{\tilde{q}}}_{1}\delta {{\tilde{q}}}_{1}\big>+\big<\delta {{\tilde{q}}}_{2}\delta {{\tilde{q}}}_{2} \big>\pm \big (\big<\delta {{\tilde{q}}}_{1}\delta {{\tilde{q}}}_{2}\big>+\big<\delta {{\tilde{q}}}_{2}\delta {{\tilde{q}}}_{1}\big>\big ), \\ \big<P_{\pm }^2\big>&=\big<\delta {{\tilde{p}}}_{1}\delta {{\tilde{p}}}_{1}\big>+\big<\delta {{\tilde{p}}}_{2}\delta {{\tilde{p}}}_{2}\big>\pm \big (\big<\delta {{\tilde{p}}}_{1}\delta {{\tilde{p}}}_{2} \big>+\big<\delta {{\tilde{p}}}_{2}\delta {{\tilde{p}}}_{1}\big>\big ), \\ \big<X_{\pm }^2\big>&=\big<\delta {{\tilde{x}}}_{1}\delta {{\tilde{x}}}_{1}\big>+\big<\delta {{\tilde{x}}}_{2}\delta {{\tilde{x}}}_{2} \big>\pm \big (\big<{\delta {{\tilde{x}}}_{1}\delta {{\tilde{x}}}_{2} }\big>+\big<\delta {{\tilde{x}}}_{2}\delta {{\tilde{x}}}_{1} \big>\big ), \\ \big<Y_{\pm }^2\big>&=\big<\delta {{\tilde{y}}}_{1}\delta {{\tilde{y}}}_{1} \big>+\big<\delta {{\tilde{y}}}_{2}\delta {{\tilde{y}}}_{2} \big>\pm \big (\big<\delta {{\tilde{y}}}_{1}\delta {{\tilde{y}}}_{2} \big>+\big <\delta {{\tilde{y}}}_{2}\delta {{\tilde{y}}}_{1}\big >\big ), \end{aligned} \end{aligned}$$

where $\big <Q_{\pm }^2\big>$ and $\big <P_{\pm }^2\big>$ are the quadrature variances of total (relative) position and momentum of mechanical modes respectively, and $\big <X_{\pm }^2\big>$ and $\big <Y_{\pm }^2\big>$ are the quadrature variances of amplitude and phase sum (difference) of cavity modes respectively. Further applying the definition in Eq. (11) to Eq. (14), the expressions of the quadrature variances become

15 $$\begin{aligned} \begin{aligned} \big<Q_{\pm }^2\big>&=V_{11}+V_{33} \pm 2V_{13}, \ \ \ \ \ \ \big<P_{\pm }^2\big>=V_{22}+V_{44}\pm 2V_{24}, \\ \big<X_{\pm }^2\big>&=V_{55}+V_{77} \pm 2V_{57}, \ \ \ \ \ \ \big <Y_{\pm }^2\big >=V_{66}+V_{88} \pm 2V_{68}. \end{aligned} \end{aligned}$$

A quantum squeezed state occurs when one quadrature component of quantum noise is suppressed below the shot noise limit at the expense of its canonical conjugate variable. Utilizing the numerical solution of the Lyapunov equation, we obtain the correlation matrix elements with relations as $V_{11}=V_{22}=V_{33}=V_{44}$, $V_{55}=V_{66}=V_{77}=V_{88}$, $V_{13}=-V_{24}$ and $V_{57}=-V_{68}$. Thus, in the absence of optomechanical coupling, the correlation elements $V_{13}$ and $V_{24}$ become zero for mechanical modes bipartite system such that $\big<Q_{\pm }^2\big>=\big <P_{\pm }^2\big >=2n+1$ whereas without non-degenerate OPA, the correlation elements $V_{57}$ and $V_{68}$ become zero for cavity modes bipartite system such that $\big<X_{\pm }^2\big>=\big <Y_{\pm }^2\big >=1$. Moreover, for $T=0$K, the nanomechanical mirrors are in their ground state ($n=0$), $\big<Q_{\pm }^2\big>=\big <P_{\pm }^2\big >=1$. According to the Heisenberg uncertainty principle the inequalities $\big<Q_{\pm }^2\big>\big <P_{\pm }^2\big > \ge |\frac{1}{2}[Q_{\pm }, P_{\pm }]|^2$ and $\big<X_{\pm }^2\big>\big <Y_{\pm }^2\big > \ge |\frac{1}{2}[X_{\pm }, Y_{\pm }]|^2$ where $[Q_{\pm }, P_{\pm }]=[X_{\pm }, Y_{\pm }]=2i$. If $\big <Q_{\pm }^2\big >$ or $\big <P_{\pm }^2\big>$ is below 1 and $\big <X_{\pm }^2\big>$ or $\big <Y_{\pm }^2\big>$ is below 1, the generation of two-mode squeezing in the state of mechanical modes and cavity modes bipartite system can be realized. Consequently, one of the quadrature variances of mechanical modes and the cavity modes is below the vacuum (quantum) noise, shot-noise variance, or standard quantum level (SQL) that corresponds to $\big<Q_{\pm }^2\big> _{vac}=\big<P_{\pm }^2\big> _{vac}=\big<X_{\pm }^2\big> _{vac}=\big <Y_{\pm }^2\big > _{vac}=1$. Note that the variances of the individual quadratures $\delta {\tilde{q}}_{j},\delta {\tilde{p}}_{j},\delta {\tilde{x}}_{j}$ and $\delta {\tilde{y}}_{j}$ are above SQL^54^, indicating they are noisy. On the other hand, since the commutation relations $[Q_{\pm },P_{\mp }]=0$ and $[X_{\pm },Y_{\mp }]=0$ are satisfied, the quadrature fluctuations $Q_{\pm }$ and $P_{\mp }$ as well as $X_{\pm }$ and $Y_{\mp }$ can be determined simultaneously without uncertainty. Similarly, we obtain commutation relations $[Q^2_{\pm }, P^2_{\mp }]=0$ and $[X^2_{\pm }, Y^2_{\mp }]=0$ such that the square of quadrature fluctuations can also be determined simultaneously with infinite precision. In addition, due to the relationship among the covariance matrix elements, we obtain the quadrature fluctuations with the form of $\langle X^2_{\pm }\rangle =\langle Y^2_{\mp }\rangle$ and $\langle Q^2_{\pm }\rangle = \langle P^2_{\mp }\rangle$. Furthermore, the degree of quadrature squeezing in *dB* units is expressed as^68^,

16 $$\begin{aligned} \big<Z^2\big>(dB)=-10\log _{10}\bigg (\frac{\big<Z^2\big>}{\big <Z^2\big >_{vac}}\bigg ) \end{aligned}$$

with $Z=Q_{\pm }$ or $P_{\pm }$ representing the quadrature of two-mode mechanical oscillators, and $Z=X_{\pm }$ or $Y_{\pm }$ representing the quadrature of two-mode optical fields.

**Fig. 2:**
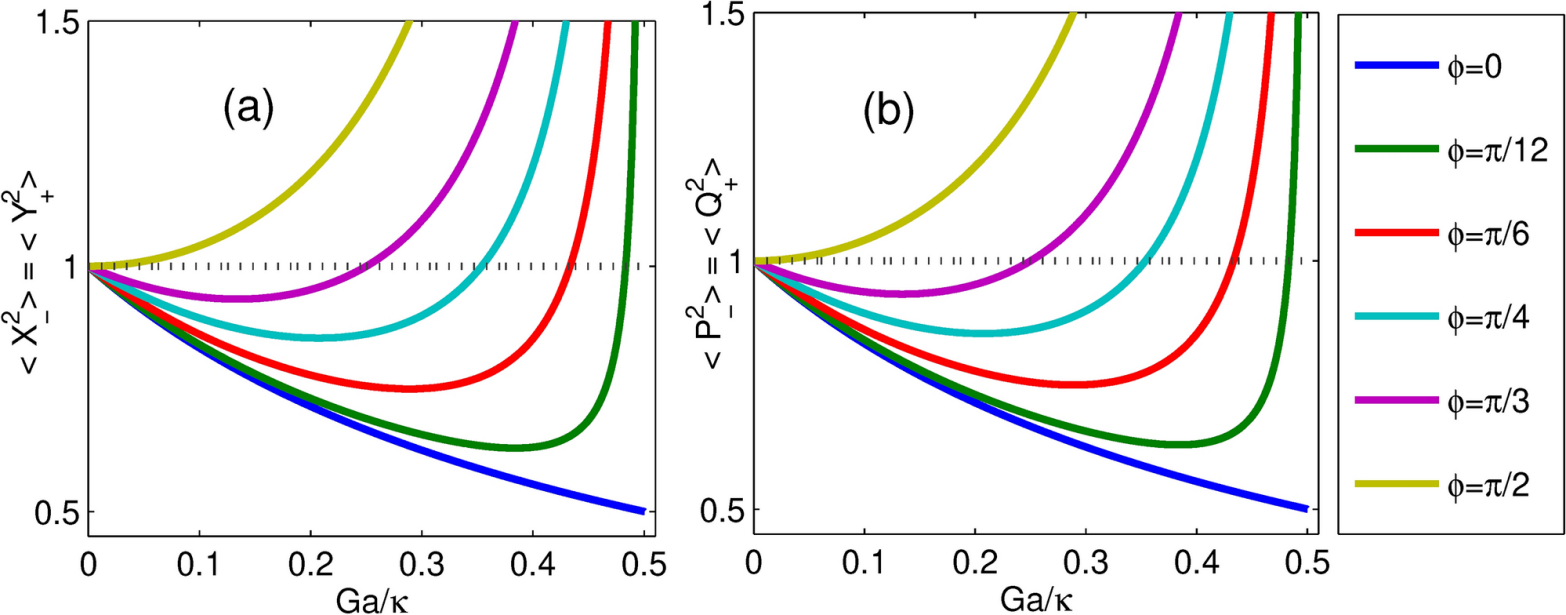
(**a**) Plots of steady-state induced quadrature squeezing in cavity modes; and (**b**) plots of the induced quadrature squeezing of mechanical modes versus normalized nonlinear parametric gain $G_a/\kappa$ for different values of parametric phases $\phi$. The temperature of mechanical baths $T= 0 K (n=0)$ and laser driving power $P = 10.7 mW$ While the other parameters are defined in the main text.

**Fig. 3:**
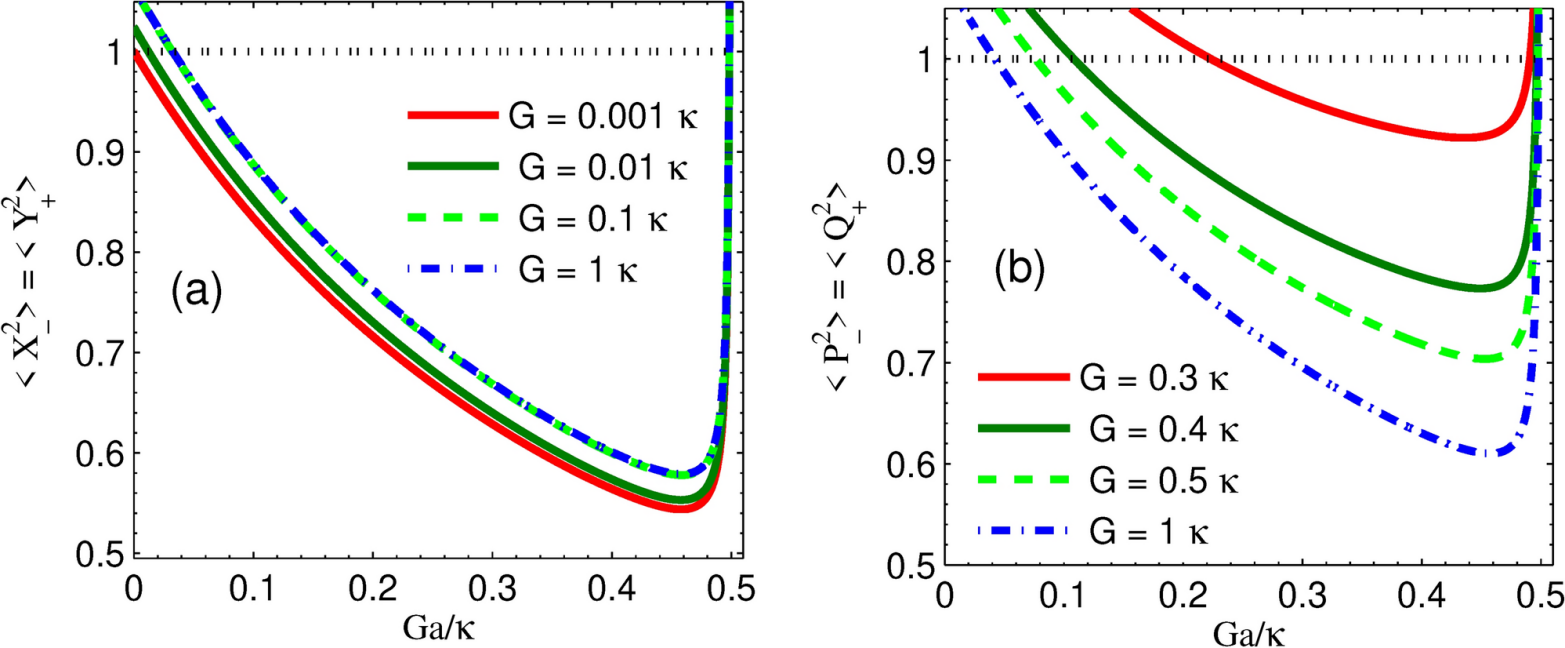
Plots of steady state quadrature squeezing (**a**) cavity modes and (**b**) mechanical modes versus normalized parametric gain for different coupling strengths for parametric phase $\phi =\pi /36$, *n* = 50 while the other parameter is the same as in Fig. 2.

Accordingly, employing the numerical solution of the covariance matrix elements of the Lyapunov equation Eq. (12) into Eq. (15), we quantify the two-mode squeezing in optical modes and mechanical modes. To generate two-mode optomechanical squeezing light, we utilize feasible recent experimental parameters used in optomechanical systems^81^. For simplicity, the parameters of the two field mirror pairs are chosen as $G_j = G$, which can be controlled by the cavity-driving input power $P_j = P$. Moreover, we take identical mechanical bath temperatures $T_j=T$ such that the thermal phonon excitation or thermal noises of mechanical modes $n_j=n$. Moreover, the laser drive frequency $\omega _{L_j}/2\pi = 2.82\times 10^{14}$ *Hz* (corresponding wave length $\lambda _{L_j}$=1064 *nm*), the frequency of the mechanical oscillator $\omega _{m}/2\pi = 947 \times 10^{3} Hz$, mass of the movable mirror $m_j= 145ng$, the initial length of the cavity $L_j=25$ *mm*, cavity decay rate $\kappa /2\pi = 215\times 10^{3}$ *Hz* $\approx 0.227\omega _{m}$, mechanical modes damping rate $\gamma _{m}/2\pi =140$ *Hz* $\approx 0.65\times 10^{-3}\kappa$ with mechanical quality factor $Q_{m}=6700$. Following this, we have investigated two-mode squeezing in the cavity and mechanical modes in the presence of non-degenerate OPA. The numerical results show that there is no squeezing related to the amplitude sum (phase difference) of quadrature variance in cavity fields, $\big<X_{+}^2\big>\ge 1, \big <Y_{-}^2\big >\ge 1$ for parametric phase $\phi \in [0,\pi /2]$. Similarly, there is no squeezing related to the total momentum (relative position) of quadrature variance for mechanical modes, $\big<P_{+}^2\big>\ge 1, \big <Q_{-}^2\big >\ge 1$ for parametric phase $\phi \in [0,\pi /2]$. Accordingly, we show that the quadrature squeezing related to the variances of the amplitude difference (phase sum), $\big<X_{-}^2\big> = \big <Y_{+}^2\big > \le 1$, of cavity modes is transferred to the quadrature squeezing of relative momentum (total position), $\big<P_{-}^2\big> = \big <Q_{+}^2\big > \le 1$, of mechanical modes for $\phi \in [0,\pi /2]$. Thus, we concentrate on discussing the squeezing in $\big<P_{-}^2\big > = \big <Q_{+}^2\big>$ and $\big<X_{-}^2\big > = \big <Y_{+}^2\big>$.

It is intriguing to investigate the effects of phase and parametric gain on optical squeezing since non-degenerate OPA is a strong contender for two-mode optical squeezing generation. Specifically, for various parametric phases, the plots of cavity field squeezing versus normalized parametric gain are displayed in Fig. 2a. It has been noticed that in the absence of the non-degenerate OPA ($G_a=0$) in the system, the optical mode is not squeezed. Nonetheless, the optical squeezing emerges when the non-degenerate OPA is incorporated into the system; it squeezes the optical modes. In particular, the optimum optical squeezing exists at $\phi =0$ for parametric gain near its threshold value. These findings suggest that the non-degenerate OPA can cause two-mode optical squeezing. Additionally, non-degenerate OPA is crucial for the two-mode mechanical squeezing existence, thus, it is imperative to look into how the mechanical squeezing relies on the parametric gain $G_a$ and phase $\phi$. Furthermore, Fig. 2b shows the various parametric phases of the mechanical modes of squeezing. From this, in the absence of the non-degenerate OPA, we also note that the two-mode mechanical squeezing is on SQL. In the presence of non-degenerate OPA, mechanical squeezing emerges for an appropriate parametric phase $\phi$. In particular, the variations in mechanical squeezing are comparable with those of optical squeezing, and the optimum degree of mechanical squeezing is almost the same as that of optical squeezing. These results show that the squeezing of the optical modes has been completely transferred to the mechanical modes due to the presence of non-degenerate OPA. Furthermore, the quadrature fluctuations in cavity modes and movable mirrors related to $\langle X^2_{-}\rangle =\langle Y^2_{+}\rangle$ and $\langle P^2_{-}\rangle = \langle Q^2_{+}\rangle$ respectively, similar to the transfer of atomic coherence quantum fluctuation features as in Ref.^82^. Moreover, it is also suggested in Ref.^83^ that two theoretical schemes with two movable mirrors in a ring geometry with three and four mirrors arrangement. The result showed that the injected amplitude squeezed light transferred to the quadrature squeezing of the relative momentum of two movable mirrors in the first scheme whereas to the total momentum of two movable mirrors in the second scheme of the movable mirrors respectively.

**Fig. 4:**
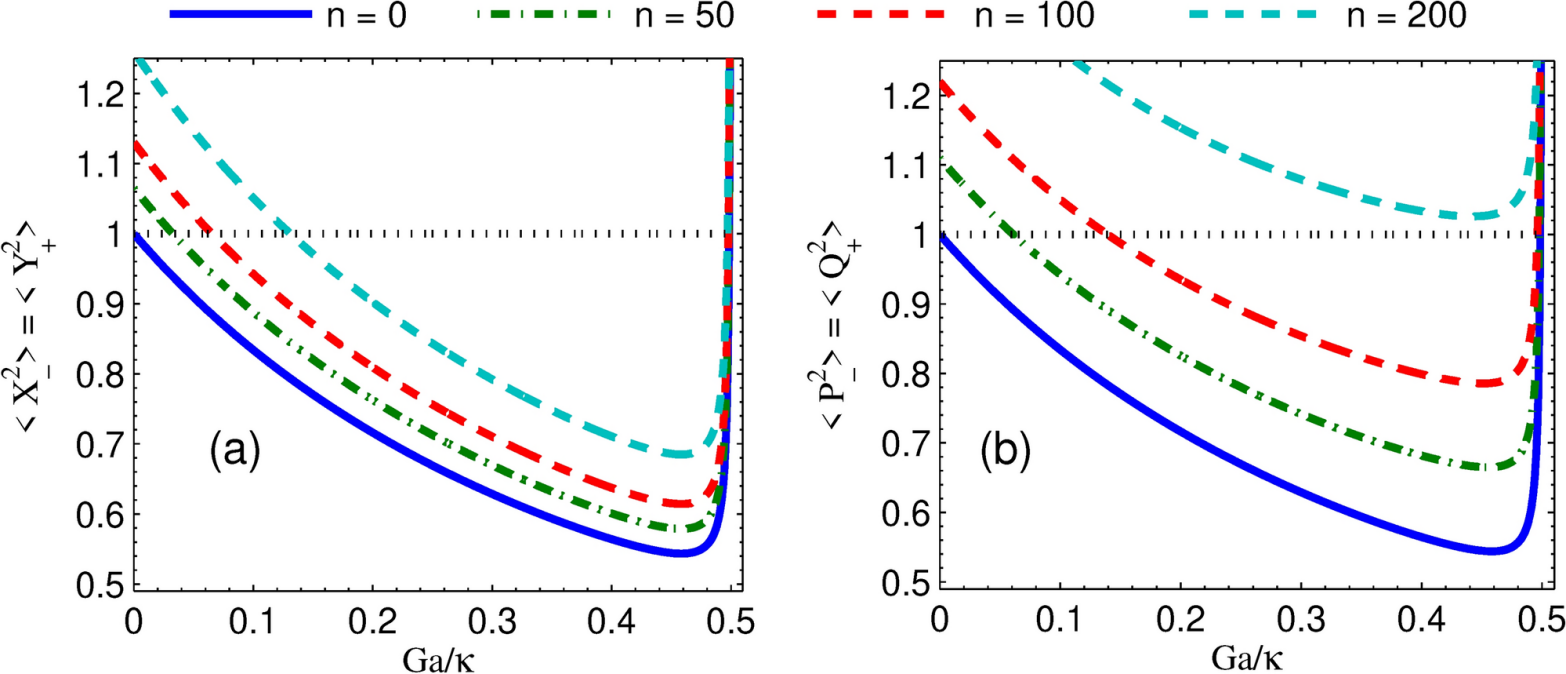
Plots of steady-state quadrature squeezing of (**a**) cavity modes and (**b**) mechanical modes versus normalized parametric gain for different thermal noises at parametric phase $\phi = \pi /36$ while the other parameter is the same as in Fig. 2.

Moreover, in Fig. 2a, b, for example, at parametric gain $0.2\kappa$, as the phase increases from $\phi = 0$ to $\phi = \pi /2$, the squeezing in both bipartite systems becomes very weak. It is worth noting that the two-mode squeezed states turn into two-mode vacuum states when $G_a$ = 0 and $\phi = \pi /2$. In the presence of non-degenerate OPA, for $\phi < \pi /2$ at each phase $\phi$, except $\phi =0$, increasing parametric gain leads to an enhancement of squeezing in both bipartite up to a particular gain corresponding to optimal squeezing; afterward, both show decreasing towards the standard quantum limit. However, at $\phi =0$ as the parametric gain increases the degree of squeezing within both the cavity modes and mechanical modes increases, up to about 50$\%$ below SQL. This result is the optimum squeezing equivalent to 3 dB.

We also study the effects of optomechanical coupling strength on quadrature squeezing, as shown in Fig. 3. Specifically, Fig. 3a shows the degree of squeezing of the two cavity modes against the normalized parametric nonlinear gain for different optomechanical coupling strengths at parametric phase $\phi =\pi /36$, *n* = 50. When the effective coupling strength changes, we find that the degree of optical squeezing is slightly reduced, and further increments above $G=0.1\kappa$ show that the degree of squeezing is not sensitive to coupling strength. Since there is an indirect interaction between the two mirrors in our setup, the effective coupling indirectly transfers the squeezing of two fields to that of two mirrors. Furthermore, the degree of squeezing of the two movable mirrors is shown in Fig. 3b. For a large effective coupling strength, we observe that the degree of squeezing has a similar path to that of the cavity modes. We demonstrate that each effective coupling strength requires a minimum parametric gain for the existence of mechanical squeezing. This implies that, in our model, there is indirect interaction between the mirrors, so we can judge that the squeezing of the two-mode fields is transferred to the squeezing of the mirrors due to the indirect coupling. Furthermore, we have shown that the optimal induced squeezing in both modes is obtained at about $G_a=0.46\kappa$ and is unaffected by field-mirror coupling strength variation. Interestingly, we show that the transfer of optical squeezing to mechanical squeezing becomes robust as effective coupling strength is enhanced. Moreover, for weak coupling strength, it is observed that the mechanical mode squeezing is above SQL, which shows that there is no squeezed light transfer to nano-mechanical oscillators.

**Fig. 5:**
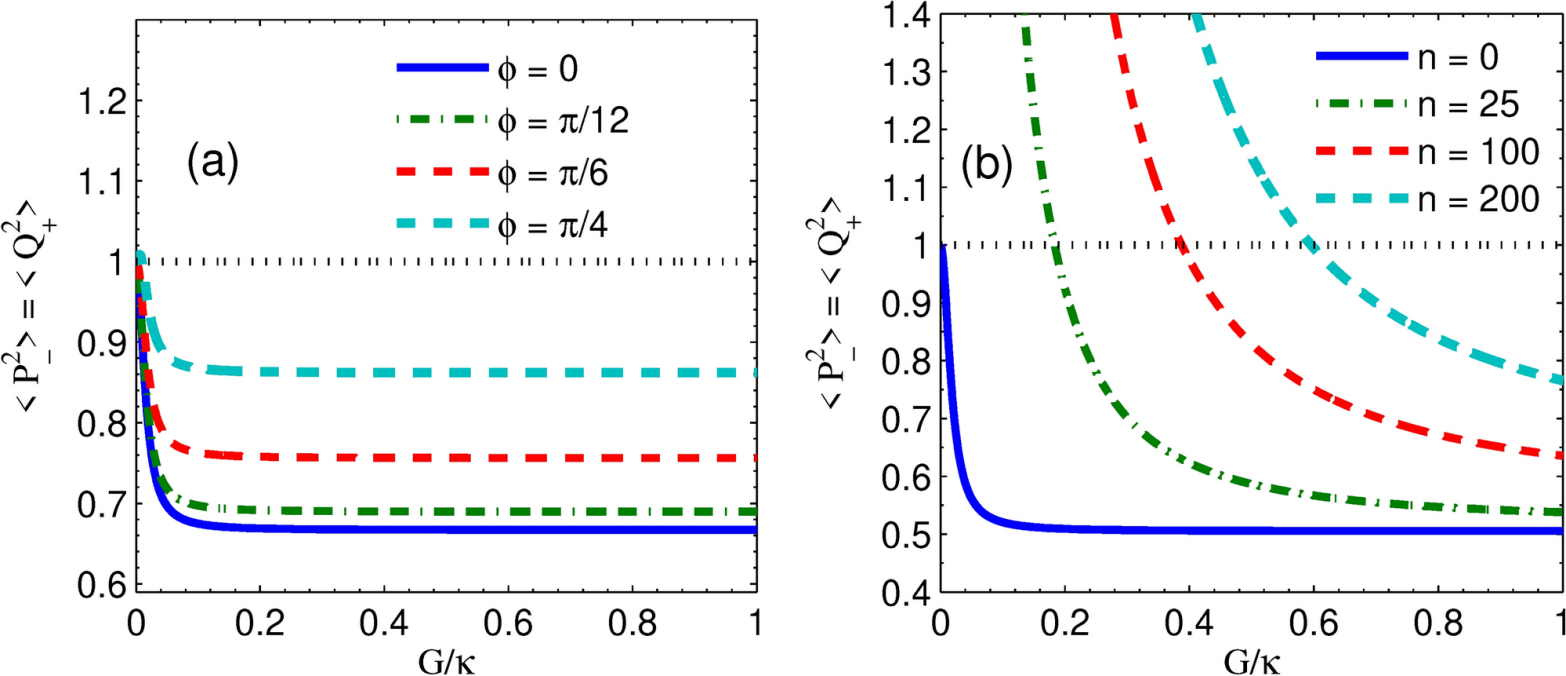
Plots steady state quadrature squeezing mechanical modes versus normalized effective coupling strength $G/\kappa$ (**a**) for different parametric phases, $n=0$, $G_a=0.25\kappa$, and (**b**) for different thermal noise of movable mirrors when parametric phase $\phi =0$, parametric gain $G_a=0.49\kappa$ while the other parameter is the same as in Fig. 2.

Another important aspect shown in Fig. 4 is the influence of thermal bath noise on the squeezing of the cavity and mechanical modes. Specifically, we observe that at very low thermal noise, the two-mode cavity field squeezing is completely transferred to mirror-mirror squeezing; however, for higher thermal noises, the transfer of squeezing efficiency becomes lower, leading the system to lose its quantum properties and behave more classically^84^. This implies that as the thermal noise of the movable mirrors increases, the degree of squeezing in both cases decreases. Furthermore, the degree of squeezing in cavity modes shows a higher sensitivity to thermal noise than power variation. However, the degree of squeezing reduction to thermal noise fluctuation is much faster in mechanical modes than in cavity modes. Interestingly, in both Figures, we show that for higher thermal noise with a lower parametric gain, squeezing in both optical fields and mechanical modes is above SQL, disrupting the coherence of optical and mechanical modes and making it harder to maintain the transfer of two-mode squeezing. It is shown that, at low thermal noise, the value of mechanical squeezing is comparable to that of optical squeezing. Thus, we suggest that to strongly enhance the squeezing to obtain squeezing in mechanical modes below SQL, stronger parametric gain is needed than squeezing in cavity modes. For thermal noise at n = 200, squeezing in mechanical modes is above SQL, even though squeezing in two-mode fields prevails. From the above results, we can say that the presence of induced squeezing in cavity modes is not necessarily a precondition for the existence of mechanical squeezing.

In Fig. 5a, it is shown that the variance of quadrature squeezing in mechanical modes versus effective coupling strength for different parametric phases with $n=0$ and $G_a=0.25\kappa$. From this, we found that as coupling strength becomes zero, the degree of squeezing corresponding to each parametric phase converges to SQL. It is also noted that for non-zero coupling strength, increasing the effective coupling strength increases the squeezing very drastically to a certain level, afterward the degree of squeezing is constant for a wide range of effective coupling strengths. Moreover, at *n* = 0 we observe that for regimes far from weak coupling, the effect of mechanical dissipation rate on squeezing is small. Furthermore, in Fig. 5b, we plot the variance of quadrature squeezing in mechanical modes versus coupling strength for different thermal noises when $\phi =0$ and $G_a=0.49\kappa$. It shows that the degree of mechanical modes squeezing increases as coupling strength is enhanced towards $G=\kappa$. Moreover, it is noted that in each of the thermal noise curves except $n=0$, there is a minimum coupling strength (minimum power) requirement for the realization of squeezing in mechanical modes. Specifically, for the thermal noise $n = 25, n=100$ and $n=200$ the corresponding minimum field-mirror coupling strengths required to initiate induced squeezing are about $0.2\kappa , 0.4\kappa$ and $0.6\kappa$ respectively. Moreover, the squeezing in mechanical modes decreases as the thermal bath temperatures of the mechanical modes increase, while the squeezing increases as coupling strength increases. Also, we observe that squeezing persists at higher temperatures for sufficient coupling strength. On the other hand, the mechanical squeezing for weak coupling regimes at low thermal noise is comparable to the squeezing for strong coupling regimes. This implies that lower laser input power is required to generate squeezing close to the optimal value.

**Fig. 6:**
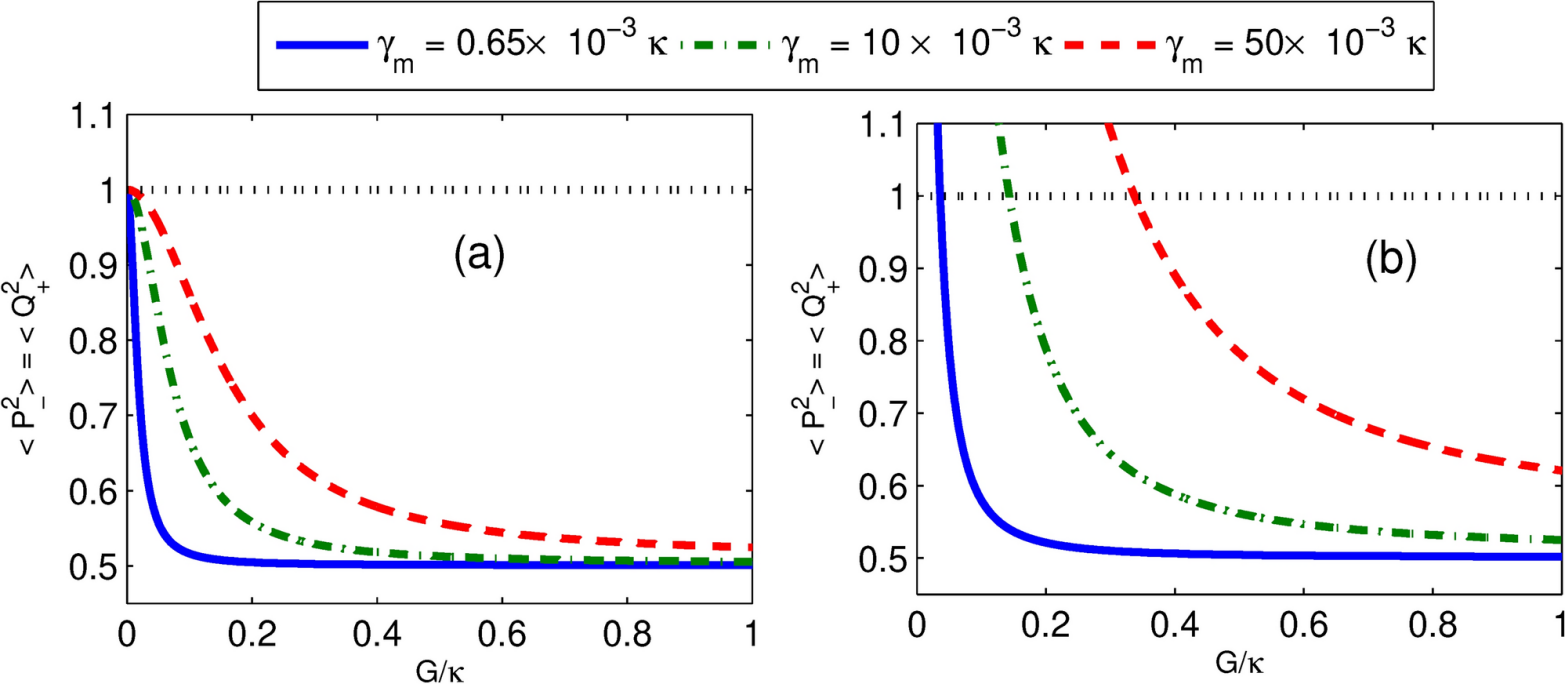
Plots of steady state quadrature squeezing in mechanical modes versus normalized optomechanical coupling strength when (**a**) $n=0$ and (**b**) $n=25$ for different mechanical damping rates at parametric gain $G_a=0.49\kappa$ and parametric phase $\phi =0$ while the other parameter is the same as in Fig. 2.

In addition, in Fig. 6, we present the effect of mechanical dissipation rate $\gamma _{m}$ on the quantum squeezing light transfer. It is well known that for a low mechanical quality factor, the mechanical dissipation can be significant, and it causes a less efficient quantum state transfer. It is shown that the mirror-mirror squeezing diminishes when the normalized mechanical dissipation rate, $\gamma _{m}/\kappa$ increases. Consequently, as the dissipation rate increases, a strong coupling is required for mirror-mirror squeezing. For example, in Fig. 6a, to achieve about 45% degree of mechanical squeezing below SQL, for $\gamma _{m}=0.65\times 10^{-3} \kappa$, $\gamma _{m}=10\times 10^{-3} \kappa$, and $\gamma _{m}=50\times 10^{-3} \kappa$, it requires coupling strength of about $G=0.05\kappa$, $G=0.2\kappa$, and $G=0.4\kappa$, respectively. This effect is similarly noticed in Fig. 6b for higher thermal noises. Moreover, the degree of squeezing decreases with increasing mechanical dissipation rates for $G/\kappa \ll 1$. In Fig. 6a, as the optomechanical strength goes to zero, the curves converge to the SQL, whereas in Fig. 6b, each of the curves corresponding to mechanical dissipation rates has its minimum coupling strength (minimum power) to display below the SQL. For the weak coupling regime for low thermal noises, there is a very drastic increment in mechanical squeezing as *G* rises. However, in this regime for high thermal noise, the squeezing in mechanical modes is above SQL. Moreover, at lower thermal noise and higher *G* values, the effect of mechanical dissipation on mirror-mirror squeezing is small. Therefore, for a given parameter of non-degenerate OPA, as the thermal decoherence rates in the mechanical baths increase, the degree of squeezing in the nano-mechanical oscillators is shown to be diminished.

Therefore, our results show the transfer of the two-mode squeezing light via a non-degenerate optical parametric amplifier in a hybrid optomechanical system. Specifically, the optimal value of two-mode optical squeezing has been achieved in a cavity containing non-degenerate OPA at zero thermal noise^85^. It is also noted that the phase-sensitive nature of non-degenerate OPA^54^ is transferred to the two correlated nano-mechanical oscillators. To beat the 3 dB limit, for example, the cavity should be coupled to biased noise fluctuation^71^ and amplitude modulated pumping field^86^. It is a realistic fact that non-degenerate OPA can induce two-mode squeezing of the optical modes when it is positioned within the optomechanical cavity. Subsequently, the two-mode squeezing in a hybrid optomechanical system can be transferred to two-mode mechanical squeezing. Thus, the physical source can be offered via non-degenerate OPA from which the two-mode squeezing in mechanical resonators is generated and transferred. Furthermore, in our scenario, the mechanical squeezing achieved is limited to the 3 dB threshold. To mitigate the effects of quantum back action and surpass this limit, it is advisable to employ additional techniques, such as quantum feedback processes^73^. In addition, we recommend utilizing both feedback mechanism^63^ and two-tone driving^87^ can effectively induce significant steady-state quantum squeezing in double mechanical quadrature resonators, exceeding the 3 dB limit.

## Detection of two-mode mechanical squeezing

In this section, we briefly discuss the detection of the generated two-mode mechanical squeezing in our scheme to be realized experimentally by utilizing the outputs of the two-mode cavity fields. This can be easily achieved in adiabatic-regime $(G \ll \kappa )$^64^ so that the cavity modes follow the dynamics of the mechanical modes and transfer their quantum state optimally^88^. Thus, we can put $\delta \dot{{\tilde{a}}}_{j}=0$ in to Eq. (7) for an adiabatic approximation and we obtain

17 $$\begin{aligned} \begin{aligned} \delta {\tilde{a}}_{1}&=\frac{2iG\kappa }{\kappa ^2-4G^2_a}\delta {\tilde{b}}_{1} -\frac{4iGG_ae^{i\phi }}{\kappa ^2-4G^2_a}\delta {\tilde{b}}^\dagger _{2} +\sqrt{\kappa }\bigg (\frac{4G_ae^{i\phi }}{\kappa ^2-4G^2_a}{\tilde{a}}^{in,\dagger }_{2} +\frac{2\kappa }{\kappa ^2-4G^2_a}{\tilde{a}}^{in}_{1}\bigg ),\\ \delta {\tilde{a}}_{2}&=\frac{2iG\kappa }{\kappa ^2-4G^2_a}\delta {\tilde{b}}_{2} -\frac{4iGG_ae^{i\phi }}{\kappa ^2-4G^2_a}\delta {\tilde{b}}^\dagger _{1} +\sqrt{\kappa }\bigg (\frac{4G_ae^{i\phi }}{\kappa ^2-4G^2_a}{\tilde{a}}^{in,\dagger }_{1} +\frac{2\kappa }{\kappa ^2-4G^2_a}{\tilde{a}}^{in}_{2}\bigg ). \end{aligned} \end{aligned}$$

Utilizing the input-output relation $\delta {\tilde{a}}^{out}_{j}=\sqrt{\kappa }\delta {\tilde{a}}_{j}-{\tilde{a}}^{in}_{j}$ , the cavity output fields take the form of

18 $$\begin{aligned} \begin{aligned} \delta {\tilde{a}}^{out}_{1}&=\frac{2iG\kappa \sqrt{\kappa }}{\kappa ^2-4G^2_a}\delta {\tilde{b}}_{1} -\frac{4iGG_ae^{i\phi }\sqrt{\kappa }}{\kappa ^2-4G^2_a}\delta {\tilde{b}}^\dagger _{2} +\frac{4\kappa G_ae^{i\phi }}{\kappa ^2-4G^2_a}{\tilde{a}}^{in,\dagger }_{2} +\frac{\kappa ^2+4G^2_a}{\kappa ^2-4G^2_a}{\tilde{a}}^{in}_{1},\\ \delta {\tilde{a}}^{out}_{2}&=\frac{2iG\kappa \sqrt{\kappa }}{\kappa ^2-4G^2_a}\delta {\tilde{b}}_{2} -\frac{4iGG_ae^{i\phi }\sqrt{\kappa }}{\kappa ^2-4G^2_a}\delta {\tilde{b}}^\dagger _{1} +\frac{4\kappa G_ae^{i\phi }}{\kappa ^2-4G^2_a}{\tilde{a}}^{in,\dagger }_{1} +\frac{\kappa ^2+4G^2_a}{\kappa ^2-4G^2_a}{\tilde{a}}^{in}_{2}. \end{aligned} \end{aligned}$$

The above equation showing that in the adiabatic regime, via the direct measurement of the output light of the cavity fields we can obtain the dynamics of the nano-mechanical mirrors. Such mathematical technique has been proposed to measure the entanglement between a cavity field and a mechanical oscillator^75^. In our case, the two-mode mechanical squeezing can be realized via a similar experimental setup developed for homodyne detection of two correlated optical modes at the output of a frequency-degenerate type-II optical parametric oscillator (OPO) below threshold^89^. This can be accomplished by varying the phases of the two local oscillators from a common reference phase that enables the measurement of the correlations between a simultaneous double homodyne detection of output fields. One can thus determine a complete measurement of the covariance matrix of such two-mode cavity output fields. Consequently, one can numerically evaluate the two-mode squeezing through Eq. (15) to evaluate the amplitude difference of the quadrature squeezing of cavity fields $\big <X^2_{-}\big>$ as a direct measure of the quadrature squeezing relative momentum $\big <P^2_{-}\big>$ of the nano-mechanical mirrors, or to evaluate the phase sum of quadrature squeezing of cavity fields $\big <Y^2_{+}\big>$ as a direct measure of the quadrature squeezing of position sum $\big <Q^2_{+}\big>$ of the nano-mechanical mirrors.

## Conclusions

In conclusion, we have studied the two-mode squeezed light transfer from optical-to-mechanical modes induced by a non-degenerate optical parametric amplifier. We consider a hybrid optomechanical system that consists of a non-degenerate OPA placed inside a doubly resonant cavity as a scheme for squeezing two-mode fields whose squeezing can be transferred to two movable mirrors through radiation pressure. We showed that when non-degenerate OPA is placed inside the optical cavity, the degree of squeezing light in both optical and mechanical modes is strongly squeezed. This leads to two-mode squeezing light being transferred into two-mode mechanical squeezing due to non-degenerate OPA under strong optomechanical coupling strength between optical and mechanical modes. Moreover, our numerical analysis asserts that it is possible to completely transfer the squeezing in cavity fields to mechanical modes for a particular selected parameter in the presence of phase-sensitive non-degenerate OPA. Consequently, both cavity fields and mechanical modes of squeezing can persist in a wide range of parametric gains of the non-degenerate OPA, with the maximum noise suppression occurring at specific parametric gains near the threshold value at zero parametric phase and in the absence of thermal noise. Moreover, we have shown that in the absence of non-degenerate OPA, cavity fields and mechanical modes of squeezing have a coherent or vacuum-state nature with no squeezed-state features. Furthermore, a particular choice of parameters related to non-degenerate OPA and weak optomechanical coupling makes the squeezing between the two mode cavity fields more robust. Thus, the squeezing in the cavity and mechanical modes degrade with increasing thermal noise or mechanical dissipation owing to enhanced decoherence rates. We also obtain that, at low thermal noise, the degree of mechanical squeezing is comparable to that of optical squeezing. Interestingly, the squeezing can be controlled reasonably by adjusting parameters such as parametric gain, phase, and laser driving power. Our scheme enables strong mechanical squeezing through the current state-of-the-art experimental parameters, and we anticipate its application in quantum sensing and quantum information processing.

## Data Availability

The datasets used and analyzed during the current study are available from the corresponding author upon reasonable request.
